# Forage quality and composition measurements as predictors of ethanol yield from maize (*Zea mays *L.) stover

**DOI:** 10.1186/1754-6834-2-5

**Published:** 2009-03-09

**Authors:** Aaron J Lorenz, Rob P Anex, Asli Isci, James G Coors, Natalia de Leon, Paul J Weimer

**Affiliations:** 1 Department of Agronomy, University of Wisconsin, Linden Drive, Madison, WI 53706, USA; 2 Department of Agricultural and Biosystems Engineering, Iowa State University, Ames, IA 50011, USA; 3 USDA-ARS, US Dairy Forage Research Center, Linden Drive West, Madison, WI 53706, USA

## Abstract

**Background:**

Improvement of biofeedstock quality for cellulosic ethanol production will be facilitated by inexpensive and rapid methods of evaluation, such as those already employed in the field of ruminant nutrition. Our objective was to evaluate whether forage quality and compositional measurements could be used to estimate ethanol yield of maize stover as measured by a simplified pretreatment and simultaneous saccharification and fermentation assay. Twelve maize varieties selected to be diverse for stover digestibility and composition were evaluated.

**Results:**

Variation in ethanol yield was driven by glucan convertibility rather than by glucan content. Convertibility was highly correlated with ruminal digestibility and lignin content. There was no relationship between structural carbohydrate content (glucan and neutral detergent fiber) and ethanol yield. However, when these variables were included in multiple regression equations including convertibility or neutral detergent fiber digestibility, their partial regression coefficients were significant and positive. A regression model including both neutral detergent fiber and its ruminal digestibility explained 95% of the variation in ethanol yield.

**Conclusion:**

Forage quality and composition measurements may be used to predict cellulosic ethanol yield to guide biofeedstock improvement through agronomic research and plant breeding.

## Background

Decreasing the cost of producing cellulosic biofuels to be competitive with gasoline and grain-based ethanol is a major goal of the US Department of Energy [1]. Three basic steps are currently used in the biochemical conversion of cellulosic biomass to ethanol: 1) physical size reduction and thermochemical pretreatment of the biofeedstock; 2) enzymatic hydrolysis of cell wall polysaccharides; and 3) fermentation of released simple sugars. The last two steps, enzymatic hydrolysis and fermentation, can be combined into a single-unit operation known as simultaneous saccharification and fermentation (SSF), which avoids end-product inhibition of hydrolytic enzymes and eliminates the need for separate hydrolysis and fermentation reactors [2]. While reducing conversion costs and increasing ethanol yield per unit mass of feedstock will initially be achieved through optimizing processing tools and techniques, further cost reductions and yield increases could be attained through improving biofeedstock quality [3]. Based on current technologies and the biological platform for producing cellulosic biofuels, increasing the polysaccharide to lignin ratio is one possible route to increasing biofeedstock quality. Exploring alternative harvesting techniques, agronomic practices, and on-farm storage methods, as well as developing high-quality crop varieties through plant breeding and transgenic approaches, are ways biofeedstocks could be improved to increase ethanol yield per dollar spent on biofeedstock production, thermochemical pretreatment, and enzymatic hydrolysis.

Experiments to study and accomplish biofeedstock improvement often generate large numbers of samples needing quality evaluation. This is especially true for plant breeding projects, where sample numbers greater than 1000 per season are typical. Standard methods for measuring ethanol yield after pretreatment and SSF [4] are laborious and impractical for evaluating large numbers of samples, but alternative assays have been developed that are more rapid and suitable for analyzing moderately large sample sets [5,6]. Normally these assays mimic the aforementioned steps of cellulosic ethanol production, and then measure either released glucose and xylose, ethanol concentration after fermentation, or both. An alternative to this approach is to determine sample composition, especially structural carbohydrate and lignin concentration, and use this information to predict relative performance for realizable ethanol yield. The National Renewable Energy Laboratory (NREL; Golden, CO, USA) developed a near-infrared spectroscopy (NIRS) calibration that provides predictions of constituent concentrations in maize stover [7,8]. As no wet chemistry is required for samples that are spectrally within range of the calibration set, this method has the advantage of being much faster and cheaper than methods involving wet chemistry. However, variation in structural composition has yet to be linked to feasible ethanol yield via pretreatment and SSF.

The plant cell wall is the primary energy source for ruminant animals, and organisms in rumen fluid face barriers to accessing cell wall carbohydrates that are similar to those experienced in SSF procedures. Therefore, forage quality assays used in ruminant nutrition are also potentially useful as primary screens in evaluating biofeedstock conversion potential. Ruminal microorganisms produce gas as they degrade forages. Weimer et al [9] showed that gas production measurements obtained from an *in vitro *ruminal fermentation (IVR) assay were correlated with bench-scale SSF ethanol measurements. However, the SSF procedure used by Weimer et al [9] was not preceded by a chemical pretreatment, which could alter the availability of structural carbohydrates. *In vitro *true digestibility (IVTD) is similar to IVR, except digestibility is determined by dry matter disappearance after a 48-hour incubation period with rumen fluid [10]. Other forage quality measurements, such as neutral detergent fiber (NDF) and acid detergent lignin (ADL), involve the detergent system methodology of Van Soest [11]. NDF is defined by insolubility in neutral detergent and approximates total cell wall concentration, especially for purposes of ruminant nutrition. NDF can be combined with IVTD to calculate NDF digestibility (NDFD) [11,12]. This quantity represents availability of cell wall carbohydrates and may be particularly relevant to the convertibility of biofeedstock to biofuels. ADL is measured as the insoluble organic matter after extraction with acid detergent solution and 72% sulfuric acid. These methods are well established and are routinely used by forage quality laboratories and research programs. Also, protocols have been automated and simplified by companies such as ANKOM Technology (Macedon, NY, USA) [13], measurements are easily calibrated with NIRS [8,14], and there is a wealth of historical data [15,16]. For these reasons, it would be very advantageous if forage quality measurements were predictive of ethanol yield using chemical pretreatment and SSF.

One way to compare potential measurements of biofeedstock quality is to utilize genetic variation for cell wall digestibility and composition. It is well known that natural genetic variation for ruminal digestibility exists within crop species [17], which is often associated with variation in total lignin concentration and ferulate cross-linking [18]. Down-regulation of key enzymes in the lignin biosynthetic pathway has been shown to produce alfalfa (*Medicago sativa*) plants with greater saccharification efficiency [19]. Also, mutations conferring lesions in the lignin biosynthetic pathway, such as the brown-midrib mutants of maize and sorghum (*Sorghum bicolor *L.), have been discovered and are well studied [20,21]. Cell walls of plants carrying the *brown-midrib3 *(*bm3*) allele typically have greater ruminal digestibility [22] and produce more glucose after enzymatic saccharification [23,24].

It was our objective to compare compositional and forage quality measurements with a rapid SSF assay preceded by chemical pretreatment. We used stover samples obtained from 12 maize varieties selected to be diverse in stover composition and digestibility. Our findings will help determine the feasibility of accurately ranking cultivars or agronomic treatments for SSF ethanol yield using high-throughput methods and without the apparatus for SSF-type assays.

## Results and discussion

Most measurements in this study can be classified into two types: convertibility and structural carbohydrate concentration. Convertibility-type measurements – ADL, lignin, IVTD, NDFD – are related to cell wall digestibility and thus availability of cell wall carbohydrates. Carbohydrate concentration measurements – glucan, xylan, NDF – relate to total cell wall concentration and thus the total amount of cell wall carbohydrates that could be converted into ethanol. All measurements were compared against ethanol yield measured by the pretreatment-Rapid SSF assay, which should reflect both convertibility and structural carbohydrate concentration. Actual convertibility achieved using Rapid SSF was calculated as the percentage of glucan converted into ethanol. IVR fermentation also reflects both types of measurements since the amount of gas produced upon ruminal fermentation is directly proportional to amount of carbohydrate accessed and fermented by the rumen microflora [10].

The range of each measurement as a percentage of the mean was at least 17% (NDF) and at most 87% (ADL) (Additional file 1). Because the fermentative organism (*Saccharomyces cerevisiae*) used in the Rapid SSF assay could only utilize six-carbon sugars, we will concentrate discussion on glucan content. There was more variability among varieties for convertibility than for glucan concentration and, therefore, Rapid SSF ethanol yield was mainly a function of convertibility (Additional files 1 and 2). The clearest examples are the *bm3 *varieties, which were lowest for glucan, but highest for convertibility and ethanol yield (Additional file 1). The *bm3 *varieties were also highest for IVR, IVTD, and NDFD and were lowest for ADL, lignin, and NDF. The variety with the lowest ethanol yield, W64A × A619, was average for convertibility and below average for glucan concentration. The combination of average convertibility and below average glucan concentration was likely responsible for its poor ethanol yield.

**Figure 1:**
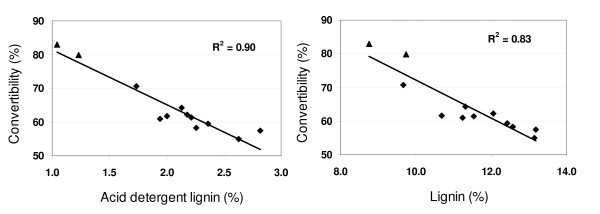
**Convertibility versus acid detergent lignin and total lignin**. Convertibility is the percentage of glucan converted to ethanol in the Rapid SSF assay. Points represent variety (*n *= 12) means. Varieties carrying the *brown-midrib-3 *mutation are symbolized by triangles.

There was no relationship between glucan concentration and ethanol yield, probably because of the negative correlation between glucan and convertibility (Additional file 2). Convertibility values in this study were lower on average than some convertibility values previously reported in the literature [25] and it is unknown how higher average convertibility would impact variability in ethanol yield and its relationship to glucan, convertibility, or other measurements. As expected, there was a strong negative correlation between convertibility and both ADL and total lignin (Figure 1). Although convertibility of glucan will improve as pretreatment and hydrolysis technology improves, developing biofeedstocks with inherently higher convertibility will decrease processing costs and will improve cellulosic ethanol economics.

Rapid SSF ethanol yield was highly correlated with forage quality methods IVR, IVTD, and NDFD (Additional file 2). These methods, which are commonly used in ruminant nutrition research and easily performed and calibrated with NIRS [18,19], may be well-suited for determining biofeedstock quality. We expected cell wall digestibility (NDFD) to be a better predictor of SSF ethanol yield than dry matter digestibility (IVTD) because the majority of carbohydrates converted to ethanol in this process are derived from the cell wall. We did observe a slightly greater correlation between NDFD and ethanol yield compared with that between IVTD and ethanol yield.

**Figure 2:**
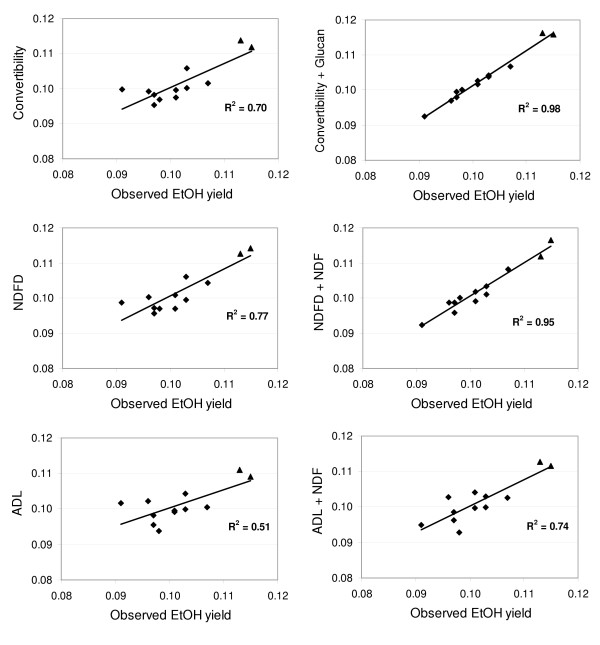
**Scatter plots for a subset of the regression models listed in Additional file **3. Ethanol yield predicted by the explanatory variables labeling the *y*-axis are plotted over observed ethanol yield from maize stover samples treated with the pretreatment-Rapid SSF assay. Regressions were performed on variety means (*n *= 12). Units for both axes are g ethanol per g dry maize stover. Varieties carrying the *brown-midrib-3 *mutation are symbolized by triangles.

Convertibility was not a perfect predictor of ethanol yield (Figure 2), suggesting that variation in carbohydrate quantity is partially influencing ethanol yield. Linear regression models including convertibility, NDFD, ADL, or lignin were compared with models that included any one of the aforementioned variables in addition to either glucan or NDF concentration (Additional file 3). Models that included both types of measurements were superior to simple linear regression models in explaining variation in Rapid SSF ethanol yield (Additional file 3). With the exception of the lignin + glucan model, the regression coefficients of glucan and NDF were all significant and positive when combined with convertibility, NDFD, or ADL. The positive regression coefficients for glucan and NDF in the multiple regression models contrast with the negative correlation coefficients observed between these variables alone and ethanol yield (Additional file 2), and suggest that greater structural carbohydrate concentrations increase ethanol yield when variation in convertibility is controlled. The NDFD + NDF model performed well (Figure 2), explaining 18% more variation than NDFD alone. While models including NDFD were superior to those including ADL, the regression coefficients in the ADL + NDF model were both significant, explaining 74% of the variation. Therefore, when NDFD measurements are difficult to obtain, ADL could adequately serve as a substitute. It should be noted that ADL measurements are known to substantially underestimate total lignin content (Additional file 1, [26]). However, our results suggest that it is a better predictor of convertibility (Figure 1) and ethanol yield (Additional file 3) than total lignin.

## Conclusion

Improvement of biofeedstock quality would increase cellulosic ethanol yield and improve profitability at a given feedstock price. Variation in biofeedstock composition and quality can be evaluated in agronomic research and breeding programs with high-throughput methods, including NIRS prediction of composition and forage quality. Our results indicate that Rapid SSF ethanol yield is mostly a function of carbohydrate convertibility, and that the contribution from glucan concentration is relatively minor. Convertibility and ethanol yield are highly correlated with forage quality measurements such as IVR fermentation, IVTD, and NDFD. Convertibility was strongly and negatively influenced by lignin content. Glucan or NDF concentration were also negatively correlated to convertibility, but when these variables were included in multiple regression models along with convertibility, NDFD, or ADL, their coefficients were significant and positive. This indicates that greater structural carbohydrate content increases Rapid SSF ethanol yield when variability in convertibility is controlled. Including glucan and NDF concentration in models along with convertibility-type measurements will increase the accuracy of biofeedstock quality predictions using surrogate measurements. More data are needed to firmly establish these relationships and build comprehensive models to predict biofeedstock quality from inexpensive and rapid measurements of digestibility and composition. The research presented here is a preliminary step in establishing such models and indicates forage quality assays are likely to be useful for evaluating biofeedstock quality.

## Methods

### Varieties and sampling

Stover samples were obtained from a set of maize varieties that were diverse for stover composition and digestibility (Additional file 1). Two varieties (entries 2 and 3) carried the *bm3 *allele. The *bm3 *allele used was from University of Wisconsin-Madison genetic stocks and is not otherwise of known origin. The non-mutant isogenic variety, W64A × A619, was also included (entry 1). The Wisconsin Quality Synthetic (WQS C3 Syn2) is a breeding population that has been improved for silage quality [14]. Entries 5 to 10 are hybrids including one parent bred for silage production, and entries 11 and 12 are hybrids bred for grain production. The 12 varieties were grown in two-row field plots as part of a larger experiment during 2005 and 2006 [8]. Plots were arranged in a randomized complete block design with three replications at two locations (Madison and Arlington, WI, USA). The planting density was 79,040 plants per hectare. One stover sample (~500 g) was obtained from each plot at grain harvest. Samples were dried at 55°C for 7 days and ground with a hammer mill to pass a 1 mm screen. An equal weight from each of the six samples in each year (three replications × two locations) was bulked to form 24 composite samples (12 varieties and two years).

### Rapid SSF analysis

Five hundred mg of ground stover sample was soaked in 30% aqueous ammonia for 24 h at room temperature and atmospheric pressure inside heat-sealed ANKOM F57 filter bags (ANKOM Technology, Macedon, NY, USA). After soaking, bags were washed with deionized water until ammonia odor was eliminated. Washed filter bags with stover samples were loaded into 25 ml Bellco DeLong flasks (Bellco Glass Inc, Vineland, NJ, USA) along with 1% (w/v) yeast extract, 2% (w/v) peptone and 0.05 M citrate buffer (pH 4.8). The total working volume was 10 ml. After sterilization, cellulase enzyme, Spezyme CP (60 filter paper units per ml; Genencor, Palo Alto, USA), and *Saccharomyces cerevisiae *D_5_A were added to the flasks, followed by incubation at 35°C for 24 h while rotating at 170 rpm. Fermentation samples were analyzed for ethanol by HPLC (Varian ProStar 210) equipped with A Bio-Rad 87 H column. SSF experiments were performed in triplicate. A more detailed description of this method is given in Isci et al [5]. Ethanol yield from each sample was expressed on a g ethanol/g (dry) maize stover. Convertibility was calculated as the percentage of glucan converted to ethanol based on a theoretical ethanol yield of 51 g ethanol per 100 g of glucose for yeast:

$$Convertibility\;(\%)=\frac{Ethanol\;produced\;(g)\;in\;reactor}{Initial\;sugar\;(glucan,g)\;in\;reactor\times 0.51}\times 100$$

The maximum value of convertibility is 100%.

### Forage quality methods

NDF and ADL were determined sequentially with the ANKOM Filter Bag method (ANKOM Technology, Macedon, NY, USA) and ANKOM-200 Fiber Analyzer. Five hundred mg of ground stover was placed in ANKOM F57 filter bags, which were heat sealed. Samples were extracted with neutral detergent, and the residue was weighed to determine percent NDF. The NDF residue was extracted with acid detergent solution, followed by extraction with 72% H_2_SO_4 _and ashing to determine percent ADL [11]. Detailed protocols can be found at the website of ANKOM Technology .

Stover IVTD is the dry matter fraction digested after a 48-hour incubation period in rumen fluid from a lactating Holstein cow and buffer solution [27]. ANKOM F57 filter bags were filled with 250 mg ground stover and incubated with rumen fluid and buffer solution in the Daisy II Incubator (ANKOM Technology, Macedon, NY, USA). After incubation, filter bags were washed with neutral detergent solution in an ANKOM-200 Fiber Analyzer to remove rumen fluid particles and non-cell wall materials. IVTD and NDF values were used to calculate NDFD by the equation: NDFD = 100{[NDF-(100-IVTD)]/NDF}. NDFD is the proportion of NDF digested during the 48-hour rumen fluid incubation.

IVR fermentation measurements were performed in duplicate on each of six samples from 2005. Incubations were conducted in nominal 60 ml serum bottles that contained 100 mg (weighed to 0.1 mg) of ground stover, 6.7 ml of Goering and Van Soest buffer [27], and 0.3 ml of cysteine-sulfide reducing agent (6.25 g/l each of cysteine HCl and Na_2_S·9H_2_O) and a CO_2 _phase. All inoculations and incubations were conducted in a 39°C room. Diluted ruminal inoculum, under continuous stirring and continuous sparging with CO_2 _in a water-jacketed vessel, was transferred by hypodermic syringe and added to the incubation bottle. Bottles were tared before adding the inoculum and weighed immediately thereafter to determine the exact amount of ruminal fluid added. Gas pressure readings were taken with a digital pressure gauge immediately after inoculum addition and after 24 h. Net gas accumulation, reported as ml gas per g of dry biomass, was calculated by subtracting the mean gas accumulation of six blank vials that contained reduced buffer and ruminal inoculum, but no biomass sample. A more detailed description of this method is given by Weimer et al [9].

### NIRS compositional analysis

Samples were scanned with a NIR Systems 6500 near infrared reflectance spectrophotometer. We used the Stover9 NIRS calibration [7,8] of NREL (Golden, CO, USA) for predictions of glucan, xylan, and lignin concentration. Samples in the Stover9 calibration set were analyzed for their structural carbohydrate and total lignin (sum of acid soluble and acid insoluble lignin) concentration using HPLC-based methods. The lignin and structural carbohydrate protocol can be found at NREL's website (see ). Stover9 calibration statistics are given in Lorenz et al [8].

### Statistical analysis

Laboratory replications were averaged and analysis of variance was conducted using the GLM procedure of SAS. For all traits but IVR gas production, linear models included variety (fixed), year (random), and variety-by-year interaction (random). Variety-by-year interaction mean squares were used as the error term for calculating significant differences between variety means. For IVR gas production, individual samples from 2005 were analyzed, and the general linear model included variety (fixed), location (random), variety-by-location interaction (random), and block nested within location (random). Differences between entries for IVR gas production were tested for significance using the entry-by-location interaction mean squares. Variability for the forage quality and composition measurements was generated by selecting maize varieties differing in these properties. Therefore, the variety source of variation was of primary interest, and the 12 entry means were used for comparing quality methods. Forage quality and compositional measurements were compared with each other and with Rapid SSF assay measurements by Pearson product-moment and Spearman rank correlations of variety means. Spearman rank correlations reflect consistency in variety rank between methods (an important criterion for plant breeders) and are less influenced by individuals at the tails of the distribution. Because IVR gas production was measured only on 2005 samples, 2006 data was excluded when comparing IVR with all other measurements. Multiple linear regression was used to determine the effect of glucan and NDF on Rapid SSF ethanol yield while controlling for variability in convertibility, NDFD, lignin, or ADL. The significance of the glucan and NDF regression coefficients, and the amount of additional variation in Rapid SSF ethanol yield explained by adding glucan or NDF to simple linear regression models including convertibility, NDFD, lignin, or ADL alone were evaluated.

## Abbreviations

ADL: Acid Detergent Lignin; IVR: *In Vitro *Ruminal Fermentation; IVTD: *In Vitro *True Digestibility; NDF: Neutral Detergent Fiber; NDFD: Neutral Detergent Fiber Digestibility; NIRS: Near Infrared Spectroscopy; NREL: National Renewable Energy Laboratory; SSF: Simultaneous Saccharification and Fermentation.

## Competing interests

The authors declare that they have no competing interests.

## Authors' contributions

AJL helped conceive and design the study, collected and processed the stover samples, made NDF, ADL, and IVTD measurements, performed the statistical analysis, and drafted the manuscript. RPA helped conceive and design the study, supervised the collection of SSF ethanol yield data, and reviewed and commented on the manuscript. AI collected the SSF ethanol yield data and drafted the Rapid SSF analysis section. JGC and NDL helped conceive and design the study, supervised sample collection and analysis, and reviewed and commented on the manuscript. PJW made IVR measurements, participated in IVTD measurements, and reviewed and commented on the manuscript. All authors read and approved the final manuscript.

## Supplementary Material

Additional File 1 **Table S1.** Variety means. Abbreviations: Rapid SSF ethanol yield (EtOH yield), percent glucan converted to ethanol (convertibility), acid detergent lignin (ADL), neutral detergent fiber (NDF), *in vitro *ruminal fermentation (IVR), *in vitro *true digestibility (IVTD), and neutral detergent fiber digestibility (NDFD). Glucan, EtOH yield, convertibility, xylan, NDF, IVR, IVTD, and NDFD variety means were ranked in descending order. ADL and lignin variety means were ranked in ascending order. Click here for file

Additional File 2 **Table S2.** Correlations. Pearson product-moment (top diagonal) and Spearman rank (bottom diagonal) correlations between biofeedstock quality, ruminal digestibility, and composition measurements. Correlations were calculated using variety means (*n *= 12). Click here for file

Additional File 3 **Table S3. **Linear regression models for predicting Rapid SSF ethanol yield. Regression was performed on variety means (*n *= 12). Click here for file
