# Supplementary material for: Forage quality and composition measurements as predictors of ethanol yield from maize (Zea mays L.) stover
Source: Biotechnol Biofuels. 2009 Mar 9;2:5. doi: 10.1186/1754-6834-2-5 (PMC2660312; doi:10.1186/1754-6834-2-5)
Supplement: Additional File 1 — Table S1. Variety means. Abbreviations: Rapid SSF ethanol yield (EtOH yield), percent glucan converted to ethanol (convertibility), acid detergent lignin (ADL), neutral detergent fiber (NDF), in vitro ruminal fermentation (IVR), in vitro true digestibility (IVTD), and neutral detergent fiber digestibility (NDFD). Glucan, EtOH yield, convertibility, xylan, NDF, IVR, IVTD, and NDFD variety means were ranked in descending order. ADL and lignin variety means were ranked in ascending order. [file 1754-6834-2-5-S1.doc]

Table 1. Variety and year means for glucan, Rapid SSF ethanol yield (EtOH yield), percent glucan converted to ethanol (convertibility), acid detergent lignin (ADL), total lignin, xylan, neutral detergent fiber (NDF), *in vitro* ruminal fermentation (IVR), *in vitro* true digestibility (IVTD), and neutral detergent fiber digestibility (NDFD). Glucan, EtOH yield, convertibility, xylan, NDF, IVR, IVTD, and NDFD variety means were ranked in descending order. ADL and lignin variety means were ranked in ascending order.

| Entry no. | Variety |  | Glucan | |  | EtOH yield | |  | Convertibility | |  | ADL | |  | Lignin † | |
| --- | --- | --- | --- | --- | --- | --- | --- | --- | --- | --- | --- | --- | --- | --- | --- | --- |
|  |  |  | % | group ‡ (rank) |  | g EtOH  g stover^-1^ | group (rank) |  | % | group (rank) |  | % | group (rank) |  | % | group (rank) |
| 1 | W64A X A619 |  | 28.9 | ef (9) |  | 0.091 | e (12) |  | 61.7 | cde (6) |  | 2.00 | bcd (5) |  | 10.7 | cd (4) |
| 2 | W64A X A619 *bm3* |  | 26.7 | g (12) |  | 0.113 | ab (2) |  | 82.9 | a (1) |  | 1.04 | a (1) |  | 8.8 | a (1) |
| 3 | F697 (*bm3*) |  | 28.1 | fg (11) |  | 0.115 | a (1) |  | 80.0 | a (2) |  | 1.23 | ab (2) |  | 9.7 | bc (3) |
| 4 | WQS C3 Syn2 |  | 28.5 | f (10) |  | 0.103 | bcd (5) |  | 70.7 | b (3) |  | 1.74 | abc (3) |  | 9.7 | b (2) |
| 5 | WQS C3 X HC33 |  | 30.7 | de (8) |  | 0.096 | de (11) |  | 60.9 | cde (8) |  | 1.95 | bc (4) |  | 11.2 | de (5) |
| 6 | W601S X LH244 |  | 32.5 | bcd (4) |  | 0.107 | abc (3) |  | 64.3 | c (4) |  | 2.13 | bcd (6) |  | 11.3 | de (6) |
| 7 | W602S X LH198 |  | 32.6 | bcd (5) |  | 0.103 | bcd (4) |  | 62.2 | cd (5) |  | 2.18 | bcd (7) |  | 12.1 | efg (8) |
| 8 | W603S X LH227 |  | 34.6 | a (1) |  | 0.097 | cde (9) |  | 54.9 | f (12) |  | 2.63 | e (11) |  | 13.1 | h (11) |
| 9 | W604S X TR7245 |  | 32.2 | bcd (6) |  | 0.101 | cd (7) |  | 61.4 | cde (7) |  | 2.22 | cd (8) |  | 11.5 | def (7) |
| 10 | W605S X HC33 |  | 31.8 | cd (7) |  | 0.097 | de (10) |  | 59.4 | ed (9) |  | 2.36 | d (10) |  | 12.4 | fghy (9) |
| 11 | DK5143 |  | 34.0 | ab (2) |  | 0.101 | cd (6) |  | 58.2 | edf (10) |  | 2.26 | cd (9) |  | 12.6 | gh (10) |
| 12 | LH227 X LH279 |  | 33.6 | abc (3) |  | 0.098 | cde (8) |  | 57.4 | df (11) |  | 2.82 | e (12) |  | 13.2 | h (12) |
|  | 2005 mean |  | 32.6 |  |  | 0.106 |  |  | 64.4 |  |  | 1.93 |  |  | 11.2 |  |
|  | 2006 mean |  | 29.7 |  |  | 0.097 |  |  | 64.6 |  |  | 2.17 |  |  | 11.5 |  |
|  | Grand mean |  | 31.2 |  |  | 0.102 |  |  | 64.5 |  |  | 2.05 |  |  | 11.4 |  |
|  | Standard error |  | 0.67 |  |  | 0.0031 |  |  | 1.43 |  |  | 0.163 |  |  | 0.33 |  |
|  |  |  | Xylan | |  | NDF | |  | IVR | |  | IVTD | |  | NDFD | |
|  |  |  | % | group (rank) |  | % | group (rank) |  | ml g^-1^ | group (rank) |  | % | group (rank) |  | % | group (rank) |
| 1 | W64A X A619 |  | 15.0 | d (11) |  | 68.3 | e (9) |  | 167 | fg (11) |  | 69.5 | c (5) |  | 55.2 | defg (8) |
| 2 | W64A X A619 *bm3* |  | 14.5 | d (12) |  | 65.6 | f (12) |  | 217 | a (1) |  | 77.1 | a (2) |  | 65.1 | a (2) |
| 3 | F697 (*bm3*) |  | 15.3 | d (9) |  | 67.4 | ef (11) |  | 202 | abc (3) |  | 77.2 | a (1) |  | 66.2 | a (1) |
| 4 | WQS C3 Syn2 |  | 15.3 | d (10) |  | 67.6 | ef (10) |  | 208 | ab (2) |  | 73.3 | b (3) |  | 60.4 | b (3) |
| 5 | WQS C3 X HC33 |  | 17.2 | c (8) |  | 71.7 | d (8) |  | 186 | de (6) |  | 68.7 | cd (6) |  | 56.3 | ed (6) |
| 6 | W601S X LH244 |  | 18.2 | abc (5) |  | 74.0 | c (5) |  | 187 | cde (5) |  | 69.8 | c (4) |  | 59.2 | bc (4) |
| 7 | W602S X LH198 |  | 18.9 | abc (4) |  | 74.8 | bc (4) |  | 195 | bcd (4) |  | 66.9 | cde (8) |  | 55.7 | def (7) |
| 8 | W603S X LH227 |  | 19.5 | ab (2) |  | 78.1 | a (1) |  | 173 | efg (10) |  | 63.2 | f (12) |  | 53.0 | g (12) |
| 9 | W604S X TR7245 |  | 17.9 | abc (6) |  | 73.5 | cd (7) |  | 177 | ef (9) |  | 68.2 | cd (7) |  | 56.7 | cd (5) |
| 10 | W605S X HC33 |  | 17.8 | bc (7) |  | 73.5 | cd (6) |  | 180 | def (7) |  | 66.2 | edf (9) |  | 54.1 | efg (9) |
| 11 | DK5143 |  | 19.3 | ab (3) |  | 76.4 | ab (3) |  | 158 | g (12) |  | 64.7 | ef (10) |  | 54.0 | efg (10) |
| 12 | LH227 X LH279 |  | 19.7 | a (1) |  | 77.5 | a (2) |  | 177 | ef (8) |  | 64.3 | ef (11) |  | 53.9 | gf (11) |
|  | 2005 mean |  | 18.5 |  |  | 74.1 |  |  | 186 |  |  | 69.6 |  |  | 59.1 |  |
|  | 2006 mean |  | 16.4 |  |  | 70.5 |  |  | -- |  |  | 68.7 |  |  | 55.9 |  |
|  | Grand mean |  | 17.4 |  |  | 72.4 |  |  | 186 |  |  | 69.1 |  |  | 57.5 |  |
|  | Standard error |  | 0.62 |  |  | 1.27 |  |  | 5.0 |  |  | 1.06 |  |  | 0.89 |  |

† Lignin values are sum of acid soluble and acid insoluble lignin.

‡ Varieties with same letter in group designation are not significantly different at 0.05 probability level.
