# Supplementary material for: Forage quality and composition measurements as predictors of ethanol yield from maize (Zea mays L.) stover
Source: Biotechnol Biofuels. 2009 Mar 9;2:5. doi: 10.1186/1754-6834-2-5 (PMC2660312; doi:10.1186/1754-6834-2-5)
Supplement: Additional File 2 — Table S2. Correlations. Pearson product-moment (top diagonal) and Spearman rank (bottom diagonal) correlations between biofeedstock quality, ruminal digestibility, and composition measurements. Correlations were calculated using variety means (n = 12). [file 1754-6834-2-5-S2.doc]

Table 2. Pearson product-moment (top diagonal) and Spearman rank (bottom diagonal) correlations between biofeedstock quality, ruminal digestibility, and composition measurements. Correlations were calculated using variety means (n = 12).

|  | Glucan | EtOH yield | Convertibility | ADL | Lignin | IVR | Xylan | NDF | IVTD | NDFD |
| --- | --- | --- | --- | --- | --- | --- | --- | --- | --- | --- |
| Glucan |  | -0.45 | -0.86** | 0.90** | 0.95** | -0.71** | 0.98** | 0.99** | -0.92** | -0.80** |
| EtOH yield | -0.32 |  | 0.84** | -0.71** | -0.60* | 0.72** | -0.36 | -0.44 | 0.74** | 0.88** |
| Convertibility | -0.80** | 0.72** |  | -0.95** | -0.91** | 0.84** | -0.80** | -0.85** | 0.97** | 0.98** |
| ADL | 0.87** | -0.49 | -0.90** |  | 0.95** | -0.74** | 0.86** | 0.91** | -0.96** | -0.92** |
| Lignin | 0.92** | -0.43 | -0.90** | 0.97** |  | -0.76** | 0.94** | 0.97** | -0.97** | -0.88** |
| IVR | -0.67* | 0.71** | 0.81** | -0.71* | -0.68* |  | -0.60* | -0.68* | 0.80** | 0.82** |
| Xylan | 0.95** | -0.18 | -0.75** | 0.86** | 0.93** | -0.51 |  | 0.99** | -0.88** | -0.74** |
| NDF | 0.99** | -0.35 | -0.82** | 0.90** | 0.95** | -0.63* | 0.97** |  | -0.92** | -0.80** |
| IVTD | -0.88** | 0.59* | 0.94** | -0.94** | -0.95** | 0.72** | -0.84** | -0.90** |  | 0.97** |
| NDFD | -0.80** | 0.72** | 0.94** | -0.89** | -0.88** | 0.81** | -0.72** | -0.83** | 0.95** |  |

* Significant at the 0.05 probability level.

** Significant at the 0.01 probability level.
