# Supplementary material for: Forage quality and composition measurements as predictors of ethanol yield from maize (Zea mays L.) stover
Source: Biotechnol Biofuels. 2009 Mar 9;2:5. doi: 10.1186/1754-6834-2-5 (PMC2660312; doi:10.1186/1754-6834-2-5)
Supplement: Additional File 3 — Table S3. Linear regression models for predicting Rapid SSF ethanol yield. Regression was performed on variety means (n = 12). [file 1754-6834-2-5-S3.doc]

Table 3. Linear regression models for predicting Rapid SSF ethanol yield. Regression was performed on variety means (n =12).

| Model † | Regression equation | R^2^ |
| --- | --- | --- |
| Convertibility | 0.0591** + 0.00066(Convertibility)** | 0.70 |
| Convertibility + Glucan | -0.0778** + 0.0014(Convertibility)** + 0.0029(Glucan)** | 0.98 |
| Convertibility + NDF | -0.096** + 0.0013(Convertibility)** + 0.0016(NDF)** | 0.95 |
| NDFD | 0.0215 + 0.00140(NDFD)** | 0.77 |
| NDFD + NDF | -0.114** + 0.00229(NDFD)** + 0.00117(NDF)** | 0.95 |
| ADL | 0.121** - 0.00967(ADL)** | 0.51 |
| ADL + Glucan | 0.0617* - 0.0219(ADL)** + 0.0027(Glucan)* | 0.69 |
| ADL + NDF | 0.0117 - 0.0240(ADL)** + 0.00192(NDF)* | 0.74 |
| Lignin | 0.135** - 0.00294(Lignin)* | 0.36 |
| Lignin + Glucan | 0.0942** - 0.00903(Lignin)* + 0.0035(Glucan) | 0.51 |

* Coefficient significant at 0.05 probability level.

** Coefficient significant at 0.01 probability level.

† Convertibility = percentage of glucan converted in ethanol with Rapid SSF assay; NDF = neutral detergent fiber; NDFD = neutral detergent fiber digestibility; ADL = acid detergent lignin; Lignin = sum of acid insoluble and acid soluble lignin.
